# *Rickettsia felis* Infection Acquired in Europe and Documented by Polymerase Chain Reaction

**DOI:** 10.3201/eid0802.010293

**Published:** 2002-02

**Authors:** Joachim Richter, Pierre-Edouard Fournier, Jasmina Petridou, Dieter Häussinger, Didier Raoult

**Affiliations:** * Universitätskliniken, Heinrich-Heine-Universität, Düsseldorf, Germany; † Centre National de la Recherche Scientifique, Faculté de Médecine, Marseille, France

We report the first case of *Rickettsia felis* infection in Europe to be documented by polymerase chain reaction (PCR) and serologic testing. *R. felis* (*1*) was first detected in 1990 as the ELB agent from the midgut epithelial cells of cat fleas (*Ctenocephalides felis*) (*2*). The pathogenic role of *R. felis* for humans has been demonstrated by its detection by PCR in five patients from Texas, Mexico, and Brazil (*3*–*5*). Following isolation of the bacterium and the first establishment of a strain in 2000, a new serologic test allowed the identification of three additional human cases (*5*).

## Case Reports

In August 2000, a 42-year-old woman and her 42-year-old husband were hospitalized in Düsseldorf, Germany, with high fever and rash of 4 and 2 days’ duration, respectively. The fever was associated with marked fatigue and headache. Four to 5 days before the onset of fever, both patients had noted a single black, crusted, cutaneous lesion surrounded by a livid halo (on the woman’s right thigh and the man’s abdomen). On admission, both patients had fever of 39ΕC and generalized maculopapular rash. The man had enlarged, painful lymph nodes in the inguinal region. Clinical examination was otherwise normal.

Laboratory investigation showed slightly elevated liver enzymes. The woman’s values were aspartate amino transferase (ASAT) 48 IU/L (normal <26); alanine amino transferase (ALAT) 29 IU/L (normal <27); gamma glutamyl transferase (g-GT) 32 IU/L (normal <200); and lactate dehydrogenase (LDH) 517 IU/L (normal <250). The man’s values were ASAT 38 IU/L, ALAT 32 IU/L, g-GT 79 IU/L, and LDH 498 IU/L. Other notable findings were elevated C reactive protein (12.8 mg/L for the woman and 11.4 mg/L for the man [normal <5]) and thrombocytopenia (93.000 x 10^9^/L) for the man. Other clinical laboratory investigations were normal. An abdominal ultrasonography showed splenomegaly in both patients.

Serologic testing for leptospirosis, as well as for other infections endemic in Germany, such as cat-scratch disease, Lyme borreliosis, ehrlichiosis, and Q fever, was negative.

The patients received doxycycline (200 mg/day) for 7 days, recovered within 3 days, and have remained well. Because symptoms resembled those of Mediterranean spotted fever, serum samples were tested for antibodies to *R. conorii;* when titers were found to be elevated, further clinical history was obtained. The patients had traveled to Costa Rica 7 months before the onset of symptoms but had not left Germany since that date. They owned two dogs, one of which had recently been adopted from an animal shelter. Neither of the dogs nor their littermates had traveled outside Germany. Both dogs, which were asymptomatic, had repeatedly had ticks and fleas, but the patients did not recall any recent arthropod bite.

Several serum samples were taken from the woman on days 4 (#1), 24 (#2), 35 (#3), and 43 (#4) and from the man on days 2 (#1) , 22 (#2), 33 (#3), and 43 (#4) after the onset of fever. A serum specimen was taken from each dog on day 35. All sera were analyzed in Marseille. Antibodies to *R. conorii*, *R. slovaca*, “*R. mongolotimonae*,” *R. helvetica*, *R. felis*, *R. typhi*, *Coxiella burnetii*, *Bartonella* *henselae*, and *Francisella tularensis* were determined by microimmunofluorescence (*6*).

Results of serologic tests were negative for *C. burnetii*, *B. henselae*, and *F. tularensis*. The woman had antibody titers to *R. felis* of 0/0 (immunoglobulin [Ig] G/IgM), 128/64, 128/64, and 128/64 for serum samples #1, 2, 3, and 4, respectively. The man had titers of 0/0, 32/16, 32/0, and 0/0 for serum samples #1, 2, 3, and 4, respectively. Cross-reactions were observed between the rickettsiae tested except for *R. typhi,* preventing the identification of the species infecting the man. A twofold difference in immunoglobulin (Ig) M titer in favor of *R*. *felis* compared with other antigens was noted for the woman. Both dogs had an IgG titer to *R. felis* of 128, but antibody cross-reactions did not allow the specific etiologic agent to be identified. A Western blot with the same antigens was performed on patient specimen #2 and the dog samples (*6*). Antibodies specifically directed at *R. felis* were observed for the woman and one of the dogs. Additionally, *R. felis* infection was confirmed by nested polymerase chain reaction (PCR) (*7*). DNA was extracted from serum #1 from both patients, taken before antibiotic therapy, and from both dog specimens with QIAGEN columns (QIAamp Tissue Kit, QIAGEN, Hilden, Germany). To avoid contamination, no positive control was used. The assay amplified from the woman’s serum a fragment of the gene encoding the PS120 protein (Figure), an intracytoplasmic protein with sequence signatures specific for most rickettsiae, including *R. felis* (*8*). The amplicons were sequenced by an ABI PRISM 310 Genetic Analyzer (Perkin Elmer, Foster City, CA). Comparison of resulting sequences to GenBank showed 100% homology with *R.* *felis*.

**Figure:**
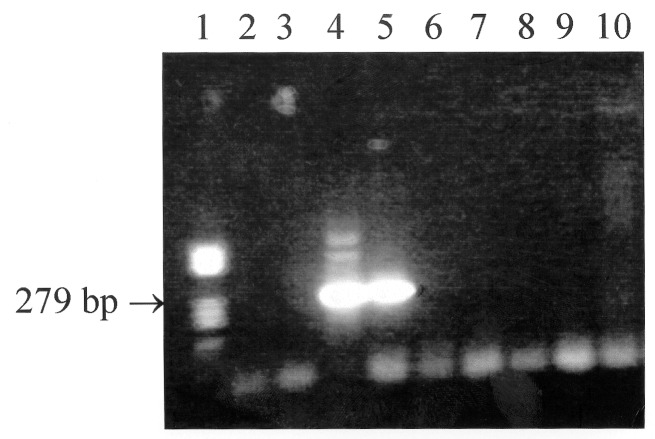
Results of the nested polymerase chain reaction (PCR) assay performed on the serum specimens from both patients and their dogs. Lane 1: standard DNA size marker V (Boehringer, Mannheim, Germany); lanes 2 and 3: serum #1 from man; lanes 4 and 5: serum #1 from woman; lanes 6 and 7: serum from dog #1; lanes 8 and 9: serum from dog #2; lane #10: negative control; lanes 2, 4, 6, and 8: pure DNA; and lanes 3, 5, 7, and 9: DNA diluted 1:10 in deionized water.

## Conclusions

Because our patients were in contact with dog ticks, a tick-borne rickettsiosis was suspected. However, no endemic tick-borne rickettsiosis has been identified in Germany to date. The most frequent rickettsiosis in Europe, Mediterranean spotted fever due to *R. conorii*, is contracted in the Mediterranean area; clustered cases, as observed for our patients, are exceptional. In contrast, African tick-bite fever, a rickettsiosis due to *R. africae,* is frequently encountered in travelers to Southern Africa (*7*). Murine typhus, caused by *R. typhi*, which has long been considered the only flea-transmitted rickettsiosis, has not been reported in Germany but is present in Southern Europe, including Spain, Portugal, Cyprus, and Greece (*9*–*12*). Until 1997, *R. felis* had only been detected in the United States. Since then, it has been detected by PCR in humans in Mexico (*4*) and Brazil (*5*) and in cat fleas from Ethiopia (*5*) and Spain (Marquez FJ, pers. comm.), thus demonstrating its presence in various areas, including the Old World, and supporting our preliminary serologic findings in French patients (*5*). In this study, serologic techniques discriminated among several rickettsiae for the woman but not her husband. Neither patient had antibodies to *R. typhi*, which suggests that antibodies to *R. felis* should be evaluated systematically in patients with typhuslike illnesses. Although no direct or indirect evidence of *R. felis* infection was obtained for the man, the simultaneous occurrence of symptoms similar to those observed in his wife strongly suggests infection with the same microorganism. Contact with fleas carried by their dogs would account for the simultaneous infection, as *R. felis* has been identified in *C. felis* fleas collected from a dog (*13*). However, neither fleas nor ticks from any of the two dogs were available at the time of examination.

Our report describes the first PCR-confirmed case of human *R. felis* infection in Europe and supports the concept that *R. felis* may be widely distributed in the Old World and should be considered in the diagnosis of typhuslike illnesses, especially following a flea bite. Further studies should be conducted to identify the vectors of this rickettsia in Europe.
